# Increased Expression of NPM1 Suppresses p27^Kip1^ Function in Cancer Cells

**DOI:** 10.3390/cancers12102886

**Published:** 2020-10-08

**Authors:** Tatsuya Kometani, Takuya Arai, Taku Chibazakura

**Affiliations:** Department of Bioscience, Tokyo University of Agriculture 1-1-1, Sakuragaoka, Setagaya-ku, Tokyo 156-8502, Japan; 44318002@nodai.ac.jp (T.K.); qrecky.tt74@gmail.com (T.A.)

**Keywords:** p27^Kip1^, nucleophosmin isoform 1, cell proliferation, CDK inhibitor, cancer cells, protein–protein interaction, mouse xenograft model

## Abstract

**Simple Summary:**

Cancer malignancy frequently correlates with a low expression of p27^Kip1^, a major cyclin-dependent kinase inhibitor, and the p27 protein level has been reportedly responsible for its antiproliferative function. However, we found the function of overexpressed p27 is suppressed in some cancer cells, suggesting that p27 function is also regulated independently of its protein level. The aim of this study was to clarify this unknown p27 regulatory mechanism and its impact on cancer proliferation. We isolated nucleophosmin isoform 1 (NPM1), which is highly expressed in variety of cancers, as a novel p27-interacting protein. Overexpressing NPM1 in normal cells suppressed and silencing NPM1 in cancer cells rescued the p27 function, respectively, in vitro. Moreover, NPM1 silencing and p27 induction in cancer cells significantly suppressed their proliferation in mouse xenografts. Our findings reveal that NPM1 is a novel p27 functional suppressor and a potential anti-cancer target, especially in cancers with normal p27 expression.

**Abstract:**

p27^Kip1^, a major cyclin-dependent kinase inhibitor, is frequently expressed at low levels in cancers, which correlates with their malignancy. However, in this study, we found a qualitative suppression of p27 overexpressed in some cancer cells. By proteomic screening for factors interacting with p27, we identified nucleophosmin isoform 1 (NPM1) as a novel p27-interacting factor and observed that NPM1 protein was expressed at high levels in some cancer cells. NPM1 overexpression in normal cells suppressed p27 function, and conversely, NPM1 knockdown in cancer cells restored the function in vitro. Furthermore, the tumors derived from cancer cells carrying the combination of p27 overexpression and NPM1 knockdown constructs showed significant suppression of growth as compared with those carrying other combinations in mouse xenograft models. These results strongly suggest that increased expression of NPM1 qualitatively suppresses p27 function in cancer cells.

## 1. Introduction

Cell cycle progression is promoted by complexes of cyclins and cyclin-dependent kinases (CDKs) and inhibited by CDK inhibitors (CKIs). The cell cycle is tightly controlled by various interactions of these factors. Disturbance of these cell cycle regulatory mechanisms can lead to carcinogenesis and cancer progression, as well as cell death [1,2].

p27^Kip1^ is a member of CKIs and was first identified as an inhibitor of the cyclin E/CDK2 complex [3]. p27 inhibits CDK activities by binding to the cyclin/CDK complex via its N-terminal domain and blocking ATP binding of CDK [4]. A major regulatory mechanism of p27 function is controlling p27 protein levels through transcriptional, translational, and post-translational regulations [5,6,7,8,9,10]. Environmental factors such as TGF-β, serum starvation, and cell contact inhibition can increase p27 protein levels [11,12].

Clinically, p27 protein levels are often low in cancer cells and there is a negative correlation among p27 protein levels and malignancy of cancer in breast cancer, lung cancer, colorectal carcinomas, and gastric carcinomas [13,14,15,16]. Previous studies on p27-null mice have indicated that p27 deficiency can cause an increase in cell proliferation, body size, and weight [17,18,19]. In addition, p27-null mice have shown higher carcinogenicity in the intermediate robe of the pituitary gland and higher tumor induction by external factors such as γ-irradiation and carcinogens than p27 wild-type mice [20].

Recent studies have indicated that abnormal localization and degradation of p27, depending on phosphorylation, suppressed p27 function and promoted tumor cell proliferation [21]. For example, serine 10 phosphorylation by UHMK1, U2AF homology motif kinase 1, promotes p27 translocation from the nucleus to the cytoplasm depending on nuclear export protein CRM1, chromosomal maintenance 1, resulting in p27 inactivation and ovarian cancer cell proliferation [7,22]. Threonine 187 is phosphorylated by CDK1/2, which promotes binding of SCF^Skp2^ ubiquitin ligase complex to p27 and its polyubiquitination and degradation [23]. Threonine 157 and 198 are also phosphorylated by PKB/AKT1, which causes 14-3-3 binding to p27, resulting in maintenance of cytoplasmic localization and degradation of p27 [24]. However, in human cancer cells, loss-of-function mutation or homozygous deletion of p27 gene is very rare [11].

On the basis of this information, normal expression level and localization are considered to be important for p27 function. However, some serious cancer patients show positive and normal expression of p27 [25], raising a question whether or not p27 function is controlled quantitatively. In this study, we focused on the antiproliferative function of p27 at normal or high expression levels in cancer cells and demonstrated that p27 did not function even under forced overexpression in some cancer cells. Thus, our experiments revealed qualitative, but not quantitative, suppression of p27 in cancer cells, and we screened for factor(s) involved in such p27 functional suppression.

## 2. Results

### 2.1. Overexpression of p27 in Cancer and Normal Cells

**Figure 1:**
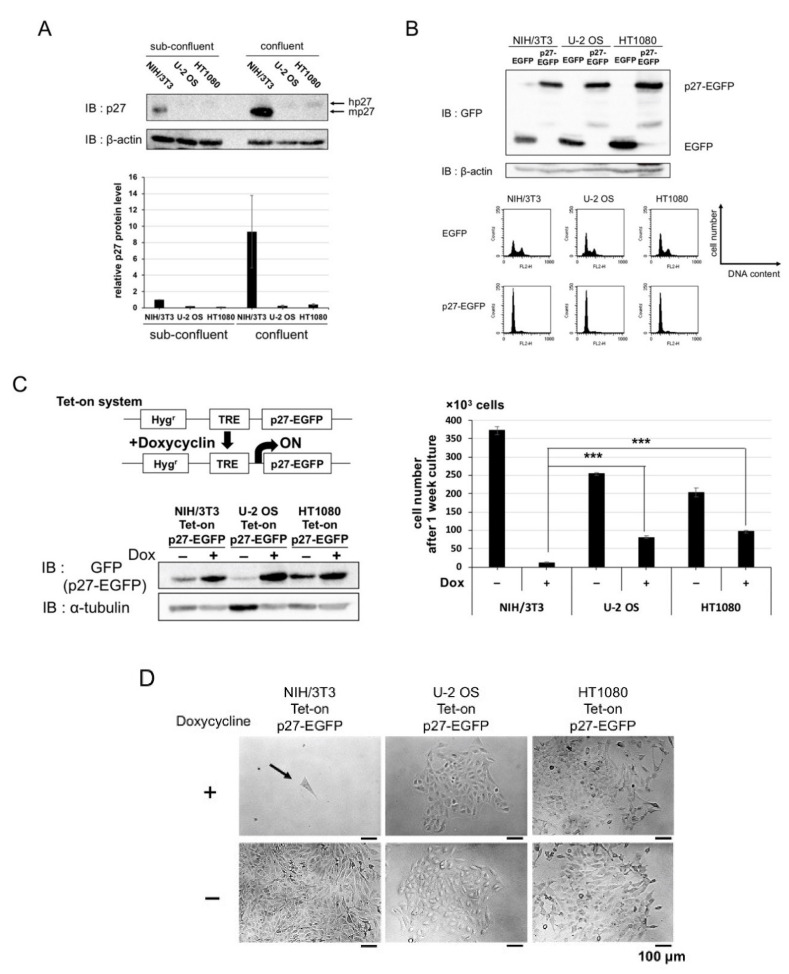
Expression of p27 and effects of p27 overexpression on proliferation in mammalian cells. (**A**) (left) p27 Expressions in murine normal cells (NIH/3T3) and human cancer cells (U-2 OS and HT1080) were detected by Western blot analysis using antibodies against p27 (hp27, human p27; mp27, mouse p27) and control β-actin. Cells were cultured under the sub-confluent and confluent conditions for three days. (right) The expression levels of p27 (relative to sub-confluent NIH/3T3) in the three cell lines were normalized by those of β-actin; (**B**) NIH/3T3, U-2 OS, and HT1080 were transfected with 10 µg EGFP or p27-EGFP expression vector. (top) Western blot detection of EGFP and p27-EGFP. (bottom) Cell cycle distributions of GFP-gated (transfected) cells were analyzed using FACS; (**C**), (left top) Construction of doxycycline (Dox)-inducible (Tet-on) p27 expression system. (left bottom) Expression of p27 in NIH/3T3, U-2 OS, and HT1080 cells carrying Dox-inducible p27-EGFP (Tet-on p27-EGFP). Cells were cultured with or without 10 µg/mL Dox for 24 h. (right) Cell proliferation assay. The three Tet-on p27-EGFP cell lines were seeded at low density (3.0 × 10^3^ cells per 35 mm dish), cultured for one week with or without 10 µg/mL Dox, and the cell number was counted (*** *p* < 0.005); (**D**) Images of cells cultured with or without Dox for one week as in (**C**). Scale bars, 100 µm.

A widespread idea is that there is a negative correlation between cancer malignancy and p27 protein expression under physiological conditions, i.e., protein expression of p27 is at a low level in cancer cells. Thus, we checked the protein expression of p27 in mammalian cells. The cell lines we used were U-2 OS (human osteosarcoma), HT1080 (human fibrosarcoma) as cancer cell lines, and NIH/3T3 (mouse fibroblast) as a normal cell line. Protein expression of p27 in cancer cells was lower than that in normal cells, either under sub-confluent or confluent condition (Figure 1A).

Firstly, to examine the effect of p27 overexpression on the cell cycle, EGFP-tagged p27 (p27-EGFP) was transiently transfected into these cell lines. Transient overexpression of p27 caused G1 phase arrest in both of the cancer and normal cell lines (Figure 1B). Secondly, we constructed those cell lines carrying doxycycline (Dox)-inducible p27, using the retroviral Tet-on system, and carried out a proliferation assay under Dox-induced p27 expression (Figure 1C,D). The induction of p27 expression inhibited the cell proliferation in normal cells but to a significantly lesser extent in the cancer cells. These results indicate that p27 is qualitatively suppressed in cancer cells.

### 2.2. Screening for Qualitative Functional Suppressor(s) of p27

**Figure 2:**
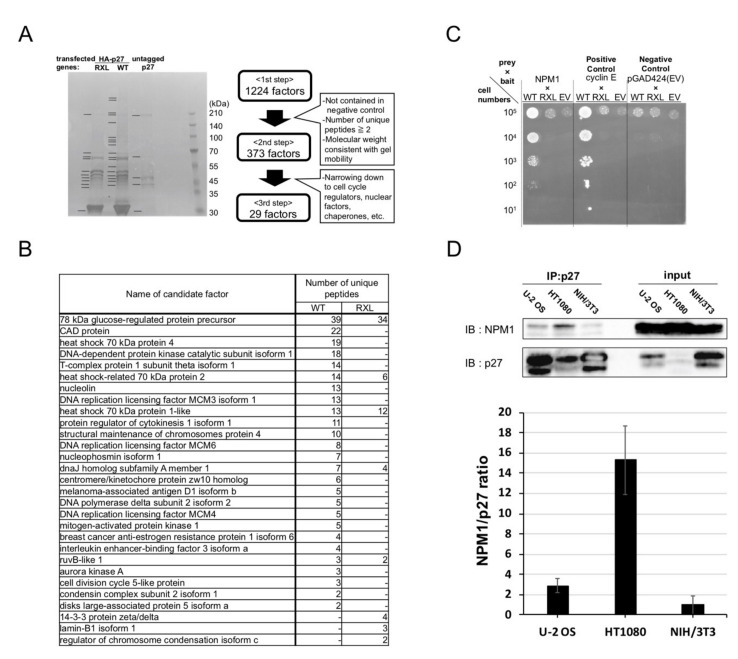
Screening for p27-interacting factors. (**A**) (left) CBB-stained SDS-PAGE gel image of anti-HA-immunoprecipitated lysates of the HEK293 cells transfected with HA-p27 wild type (WT, middle), HA-p27 RXL mutant (RXL, left), or untagged p27 (right). Protein extracts, from the bands marked with black bars, were subjected to mass spectrometry (MS). (right) Screening process for the candidate factors interacting with p27; (**B**) List of 29 candidate factors for interacting with p27 WT or RXL after the screening. Number of unique peptides identified in each of the MS samples is shown in the right columns; (**C**), Yeast two-hybrid assay between p27 and its interacting factors. PJ69-4A cells carrying the combinations of vectors expressing bait proteins (WT, p27 wild type; RXL, p27 RXL mutant; and EV, empty vector) and prey proteins (NPM1, nucleophosmin isoform 1; cyclin E; pGAD424, empty vector) were spotted onto the selection plate with five-step dilutions; (**D**) U-2 OS, HT1080 and NIH/3T3 cells carrying Dox-inducible p27 were treated with 10 µg/mL Dox for 24 h before harvest. The cell lysates were subjected to anti-p27 immunoprecipitation and Western blot analysis. Ratios of NPM1 protein amount co-immunoprecipitated with p27 protein are plotted in the right (relative to that in NIH/3T3 cells).

p27 negatively regulates the cell cycle by binding the cyclin/CDK complex and inhibiting CDK activity. Therefore, the qualitative suppression of p27 in those cancer cells could be caused by the factor(s) preventing p27 from binding cyclin/CDK. We hypothesized that such qualitative functional suppressor(s) interact with p27 and carried out the screening for p27-interacting factors. HEK293 cells, which show high efficiency for transfection, were transfected with HA-tagged p27 wild type (HAp27 WT), untagged p27 WT as a negative control, and HAp27 RXL mutant (RXL), which could not bind to cyclins due to mutations in its cyclin-binding motif. Then, the cell extracts were subjected to anti-HA immunoprecipitation and SDS-PAGE. Figure 2A shows the CBB-stained gel. The protein bands, marked with black bars, were sliced and subjected to mass spectrometry. Firstly, 1244 factors were detected as candidate factors interacting with p27. Secondly, these were narrowed down to 373 factors by the following criteria: not in negative control, containing two or more unique peptides, and their detected molecular weights were consistent with the gel mobility. Finally, by focusing on cell cycle-related factors other than cyclins and CDKs, nuclear factors, and chaperones among the 373 factors, the 29 candidate p27-interacting factors were selected (Figure 2B).

### 2.3. Nucleophosmin Isoform 1 (NPM1) Interacts with p27

To efficiently perform interaction analyses among p27 and these 29 factors, we carried out a yeast two-hybrid assay. The p27 WT, RXL as baits, and the candidate p27-interacting factors as preys were introduced into yeast PJ69-4A cells. Complementary DNAs of the candidate factors were derived from U-2 OS cells. The PJ69-4A cells introduced with p27 WT and cyclin E (a positive control for p27 interaction) grew on selection medium, verifying the experimental system (Figure 2C). Among those candidate factors, only nuceleophosmin isoform 1 (NPM1, also known as nucleolar phosphoprotein B23, No38, and numatrin) [26] was shown to interact with p27 WT but not with RXL mutant (Figure 2C). This suggests that NPM1 interacts with p27 through the cyclin-binding motif in p27, and thus NPM1 competes with cyclins for binding to p27.

Next, we tested the interaction between p27 and NPM1 in mammalian cells by co-immnoprecipitation analysis. Cell lysates of NIH/3T3, U-2 OS, and HT1080 cells carrying Dox-inducible p27 and treated with Dox were subjected to anti-p27 immunoprecipitation and SDS-PAGE and Western analysis. Figure 2D shows that NPM1 was co-immunoprecipitated with p27 in mammalian cells, demonstrating that NPM1 interacts with p27, and indeed, more strongly in cancer cells than in normal cells. Immunoprecipitation using control rabbit IgG yielded only trace amount of NPM1 and p27 proteins in the precipitates (Figure S1), indicating that NPM1 is specifically co-immunoprecipitaed with p27.

### 2.4. Increased Expression of NPM1 Suppresses p27 Function In Vitro

**Figure 3:**
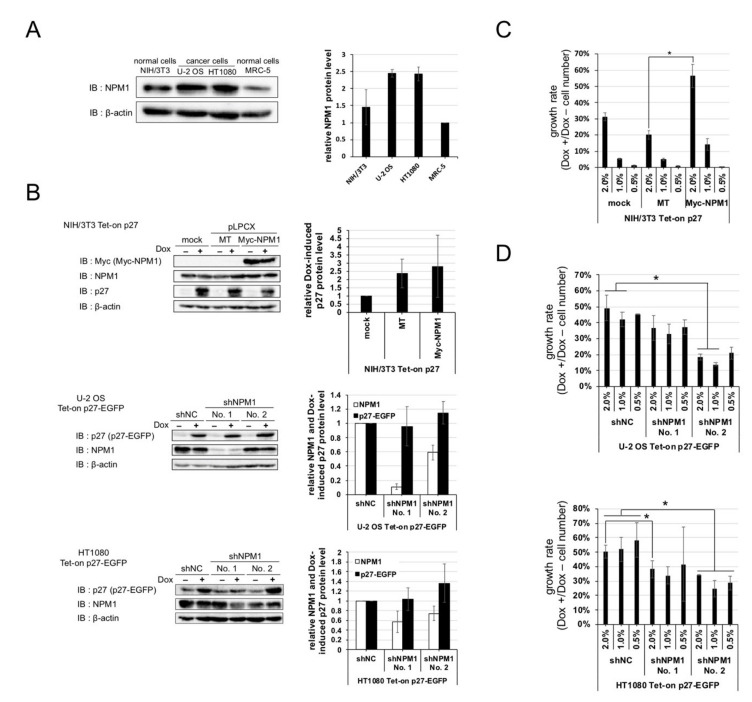
Nucleophosmin isoform 1 (NPM1) suppresses the function of p27 in vitro. (**A**) (left) Expressions of NPM1 protein in human cancer cell lines (U-2 OS and HT1080) and normal cell lines (murine NIH/3T3 and human MRC-5) were detected by Western blot analysis using antibodies against NPM1 and control β-actin. (right) The relative expression levels of NPM1 (normalized with β-actin) in three cell lines are plotted (MRC-5 = 1); (**B**) (left) Expressions of NPM1 and p27 in NIH/3T3 cells carrying Dox-inducible p27 and overexpressing NPM1 (top) and U-2 OS and HT1080 cells carrying Dox-inducible p27 and expressing NPM1 shRNAs (middle and bottom) were detected by Western blot analysis using antibodies against p27, NPM1, and control β-actin (mock, no vector; MT, Myc-tag only expression vector; Myc-NPM1, Myc-tagged NPM1 expression vector; and shNC, negative control shRNA). The relative expression levels of p27 and/or NPM1 (normalized with β-actin) in the three cell lines are plotted in the right (mock or shNC = 1); (**C**) Proliferation assay for NIH/3T3 Tet-on p27 cells with or without NPM1 overexpression. The cells were seeded at low densities (1%, 3 × 10^3^ cells per 35 mm dish), cultured for one week with or without 10 µg/mL Dox, and counted. Growth rate is the division of the Dox+ cell number by the Dox–; (**D**) Proliferation assays for U-2 OS (top) and HT1080 (bottom) Tet-on p27-EGFP cells with or without NPM1 knockdown. * 0.01 < *p* < 0.05.

NPM1 is an abundant nucleolar protein found in nucleolus in growing cells. We checked the expressions of NPM1 in normal cells and cancer cells and observed that the protein level of NPM1 is about two times higher in cancer cells than normal cells (Figure 3A).

Thus, we predicted that increased expression of NPM1 in cancer cells is involved in suppression of p27 function. To verify the effect of increased expression of NPM1 on p27 function, normal cells carrying Dox-inducible p27 and NPM1 stable overexpression constructs and cancer cells carrying Dox-inducible p27 and NPM1 stable knockdown constructs were established (Figure 3B), and their growth rates were compared. NPM1-overexpressing normal cells showed a higher growth rate than the mock-expressing normal cells upon p27 induction (Figure 3C). Conversely, NPM1 knocked down cancer cells showed a lower growth rate than the control cancer cells upon p27 induction (Figure 3D). The cell numbers, after one week of cultivation with and without Dox, showed that overexpression or NPM1 knockdown did not have significant effect on proliferation of all the cell lines in the absence of Dox (Figure S2). These results suggest that increased expression of NPM1 in cancer cells suppresses p27 function in vitro. Cancer cells, especially U-2 OS cells, carrying shNPM1 No. 1 showed a higher growth rate (i.e., stronger suppression of p27) than cancer cells carrying shNPM1 No. 2, although the former showed a lower expression of NPM1 than the latter (Figure 3B,D), suggesting that NPM1, depending on its expression level, may exert dual effects on p27 function (see Discussion).

### 2.5. NPM1 Suppresses p27 Function in Xenografted Tumors in Mouse

**Figure 4:**
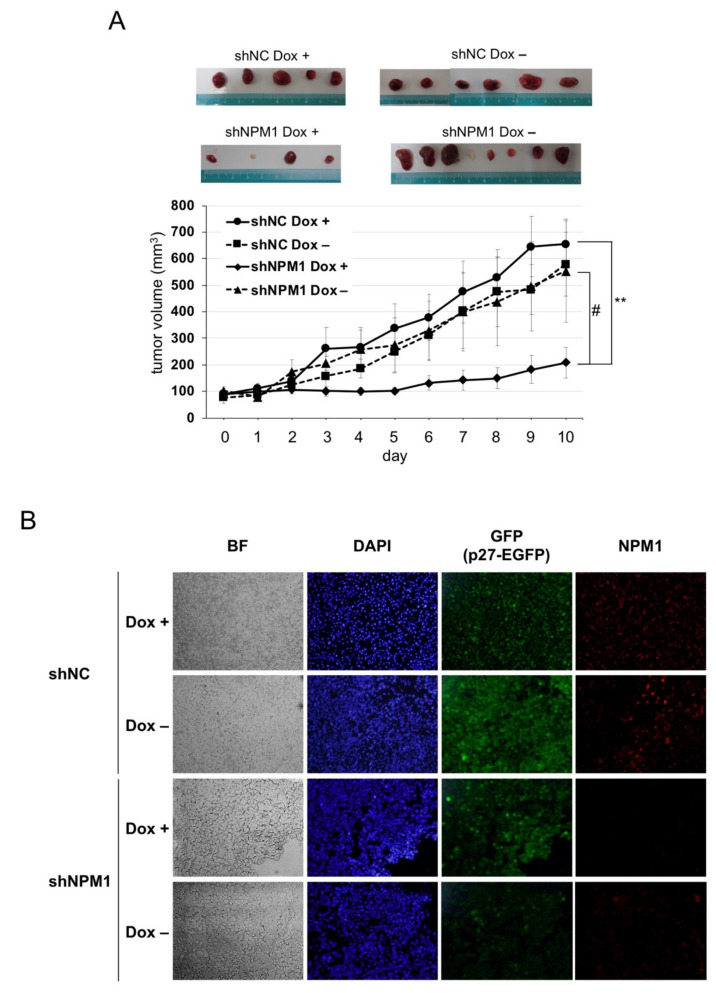
NPM1 suppresses p27 function in mouse xenograft model. (**A**) Control shRNA (NC)- and shNPM1 (No. 2)-introduced HT1080 Tet-on p27-EGFP cells were injected subcutaneously into the back of BALB/c-nu nude mice. One week after injection, mice were fed with Dox (200 µg/mL) or only solvent and the size of tumor was measured every day. (top) Samples of the tumors excised from four groups of xenografted mice. (bottom) The tumor volume was calculated as follows: volume (mm^3^) = (length, mm) × (width, mm)^2^ × 0.523. ** 0.005 < *p* < 0.01 and # 0.05 < *p* < 0.1; (**B**) Immunofluorescence analysis of p27 and NPM1 in tumors. The tumors excised from each group of xenografts were subjected to immunofluorescence analysis using anti-NPM1 antibody (red). Nuclei were stained with DAPI. BF, bright field.

Next, to verify the effect of NPM1 on p27 function in more physiological settings, mouse xenograft model experiments were carried out. The HT1080 cells knocked down for NPM1 and carrying Dox-inducible p27 were transplanted into the back of BALB/c-nu nude mice and the tumor growth was monitored with or without Dox feeding. The tumors derived from the negative control cells showed a steady growth, with or without p27 induction. By contrast, the growth of tumors derived from NPM1 knockdown cells was markedly suppressed upon p27 induction as compared with no p27 induction (Figure 4A(#)). Importantly, we observed that the combination of p27 induction and NPM1 knockdown significantly suppressed tumor growth (Figure 4A(**)).

Finally, to check whether p27 expression was induced by Dox and NPM1 was knocked down in tumors, immunofluorescence analysis was carried out (Figure 4B). After the tumors were excised from each nude mouse, their cryosections were subjected to immunofluorescence analysis. On the one hand, p27-EGFP (green) was detected in the nuclei of Dox + tumor cells, while some background whole cell signals were observed in Dox– cells (especially in negative control shRNA Dox– cells). On the other hand, NPM1 (red) was detected remarkably weaker in tumors derived from NPM1 knockdown cells than in those from negative control cells.

These results strongly suggest that the increased expression of NPM1 suppresses p27 function in vivo, as well as in vitro.

## 3. Discussion

An abnormal cell cycle causes carcinogenesis and cancer progression. CKIs including p27 are important factors involved in cell cycle regulation. The p27 functions are often suppressed by its protein degradation via phosphorylation. However, we revealed a qualitative suppression of p27 in some cancer cell lines. This qualitative suppression was not dependent on transport of p27 from nuclei to cytoplasm because p27 localization was predominantly nuclear in the cell lines we used (Figure S3). We assume that the effects of Dox-inducible p27 on cell proliferation is not very artificial because this system shows milder expression than transient transfection and some mammalian tissues maintain relatively high p27 protein expression. Therefore, employing the Dox-inducible system may have allowed us to discover the qualitative suppression of p27 function. These findings also suggest that p27 function is sensitive to its protein expression level and expression system.

We identified NPM1 as a novel p27-interacting factor by proteomic analysis and interaction analyses. We also revealed NPM1 interacted with p27 through its cyclin-binding motif by yeast two-hybrid assay. This result suggests NPM1 prevents p27 from binding to cyclins. Consistently, we observed that Dox-induced p27 protein was co-localized with transfected NPM1 in nucleoli in U-2 OS cells but not in NIH/3T3 cells (Figure S4). This result suggests that NPM1, which is known as a nucleolar protein [26], binds to p27 and separates it from nucleoplasm (where cyclin/CDK complexes are localized) to nucleoli. In addition to NPM1, we identified some candidate factors that interacted with p27. Although some of them did not show any interaction with p27 in the yeast two-hybrid assay, it could be due to a lack of post-translational modification(s) necessary for their interaction with p27. For example, 14-3-3, one of those factors isolated from our proteomic screening (Figure 2C), mainly binds to p27 phosphorylated at threonine 198 [24]. Hence, other interaction analyses may be necessary.

We suggest that the increased expression of NPM1 qualitatively suppressed p27 function in vitro and in vivo. We also speculate that the expression level of NPM1 is involved in functional regulation of p27 (Figure 3). NPM1 knockdown with shNPM1 No. 1 in HT1080 shows a significantly lower growth rate than the control (Figure 3D, at initial cell density of 2%). The NPM1 suppression level in HT1080 cells with shNPM1 No. 1 (0.57) is relatively close to that with No. 2 (0.74), which is also comparable to that in U-2 OS cells with No. 2 (0.59) (Figure 3B). Thus, apparently, a moderate NPM1 suppression level (0.6–0.7) leads to a rescue of growth inhibitory function of p27 while much stronger NPM1 suppression results in a loss of growth inhibition. Since shNPM1 No. 1 does not affect cell proliferation in the absence of Dox (Figure S2), it seems unlikely that low NPM1 level might exert other pro-proliferative effect(s) independently of p27. Thus, above a certain expression level, NPM1 suppressed p27, while at a relatively lower level, it could be necessary for p27. This is consistent with the fact that normal cells, where p27 can function normally, maintain certain levels of NPM1 (Figure 3A). Further study is necessary to clarify if p27 function depends on NPM1 expression level.

NPM1 is a nucleolar and multifunctional protein. For example, NPM1 is involved in ribosome assembly as ribonuclease, associated with centrosome duplication and controls cellular apoptotic response by inhibiting ARF (alternative reading frame) [27,28,29,30,31]. In some cancers, mutant forms of NPM1 including the one fused to ALK, anaplastic lymphoma receptor tyrosine kinase, and NPM1c+ (NPM1 cytoplasmic positive) are known [32,33]. In this study, however, qualitative suppression of p27 by increased expression of NPM1 does not associate with these NPM1 mutants since NPM1 cDNA cloned from the U-2 OS cells we used was confirmed to be wild-type. In other words, the increased expression of NPM1 should be caused by mutations of factor(s) other than NPM1 itself in U-2 OS cells. As one of such factor(s) responsible for the increased expression of NPM1, we propose ARF because ARF negatively regulates NPM1 expression [34] and its expression is not detected in U-2 OS and HT1080 cell lines. Moreover, the ARF/INK4a locus, which is overlapped with INK4 family p16 gene, is one of the most frequently deleted loci in human cancer [35]. Therefore, the qualitative suppression of p27 by NPM1 could occur in various types of cancer in which ARF deficiency leads to high NPM1 expression. We are currently analyzing the regulatory relationships among p27, NPM1 and ARF.

In summary, we identified NPM1 as a novel factor interacting with p27. The NPM1 expression level is increased in cancer cells, which qualitatively suppresses p27 function and promotes cell proliferation of cancer cells in vitro and in vivo. These findings have an impact on the conventional understanding of p27, as a novel mechanism for its functional regulation. In addition, they might contribute to cancer prevention, as well as cancer treatment, because a lot of tissue cells maintain high p27 protein expression in our body.

## 4. Materials and Methods

### 4.1. Cell Lines and Culture

NIH/3T3 cells were provided by C. Sherr (St. Jude Children Hospital, Memphis, TN, USA), and U-2 OS, HT1080, and HEK293 cells were provided by M. Ohtsubo (Beppu University, Oita, Japan). MRC-5 were purchased from RIKEN BioResource Center (Tsukuba, Japan). Cell were grown at 37 °C in Dulbecco’s modified Eagle medium (DMEM) containing 10% tetracycline-free fetal bovine serum (Takara bio Inc., Kusatsu, Japan) with 5% CO_2_.

### 4.2. Plasmids

Enhanced green fluorescent protein (EGFP) cDNA from pEGFP-C1 (Takara bio Inc.) was cloned into mammalian gene expression vector pCS2+ [36]. pCS2+p27 and pCSMT-cyclin E were provided by M. Ohtsubo. The p27 cDNA fused to N-terminal of EGFP was also cloned into pCS2+ to construct pCS2+p27-EGFP.

Doxycycline (Dox)-inducible exogenous p27 expression system was constructed using pRevTet-On and pRevTRE (Takara bio Inc., Kusatsu, Japan.). Yeast two-hybrid system was constructed using pGBTK [37] and pGAD424 (Takara bio Inc., Kusatsu, Japan) as a bait and a prey protein expression vector, respectively. Stable overexpression and stable knockdown for NPM1 were carried out using pLPCX and pSIREN Retro-Q (Takara bio Inc., Kusatsu, Japan), respectively.

### 4.3. Transfection and Flow Cytometry

Ten µg each of pCS2+p27-EGFP or pCS2+EGFP was transfected into 4 × 10^5^ cells using a modified Ca-phosphate method [38]. Sixteen hours after the transfection, cells were refed and cultured for additional 24 h. Harvested cells were fixed with 80% ethanol and stained with propidium iodide. Flow cytometry was performed, as described previously, using FACSCalibur cytometer and CELLQUEST software ver. 5.2.1 (BD Biosciences, Franklin, NJ, USA) and data were gated for GFP-positive cells [39].

### 4.4. Western Blotting

Harvested cells were rinsed once with phosphate-buffered saline (PBS) and lysed at 100 ℃ for 5 minutes in 1 × SDS sample buffer (62.5 mM Tris-HCl (pH 6.8), 2% SDS, 10% glycerol, 2.5% β-mercaptoethanol, 0.01% bromophenol blue). SDS-PAGE and Western blotting analysis were performed as described [38] using anti-p27 mouse monoclonal (clone 57, BD Biosciences) and rabbit polyclonal (clone C-19, Santa Cruz Biotechnology, Dallas, TX, USA), anti-β-actin rabbit polyclonal (Bioss), anti-NPM1 mouse monoclonal (clone FC61991, Thermo Fisher Scientific, Waltham, MA, USA) and rabbit polyclonal (Bethyl, Montgomery, AL, USA), anti-Myc-tag rabbit polyclonal (clone 562, MBL, Nagoya, Japan), and anti-α-tubulin mouse monoclonal (GeneTex, Irvine, CA, USA) antibodies.

### 4.5. Establishment of Cell Lines Carrying Dox-Inducible p27 Constructs

NIH/3T3, U-2 OS, and HT1080 cell lines carrying Dox-inducible p27-EGFP were established by retroviral transfer, as described previously [39]. First, those cell lines were infected with retroviral supernatants derived from pRevTet-On and selected with Geneticin (Thermo Fisher Scientific). Then, the stable infectants were secondly infected with retroviral supernatants derived from pRevTRE-p27 or pRevTRE-p27-EGFP, carrying human p27 or p27-EGFP cDNA, respectively, and selected with Hygromycin B (Merck, Darmstadt, Germany) to establish double infectants named as NIH/3T3 (U-2 OS or HT1080) Tet-on p27 (or p27-EGFP).

### 4.6. Cell Proliferation Assay

Cells carrying Dox-inducible p27 were seeded at low densities (1% = 3 × 10^3^ cells per 35 mm dish), refed every two days, and cultured for seven days with or without 10 µg/mL Dox. On the seventh day, cells were harvested and counted.

### 4.7. Preparation of Cell Extracts and Immunoprecipitation

Cells harvested by trypsin-EDTA were rinsed once with Tris-buffered saline (TBS, pH 7.4) and lysed on ice in RIPA buffer (10 mM Tris-HCl, pH 7.4, 0.15 M NaCl, 0.5% Nonidet P-40, 50 mM NaF, 1 mM Na-vanadate, 10 µg/mL each of aprotinin, leupeptin, and pepstatin A). Immunoprecipitation was performed, as described in [38], using anti-HA agarose beads (Thermo Fisher Scientific), anti-p27 (C-19) rabbit polyclonal antibody (Santa Cruz Biotechnology), or control rabbit IgG (MBL).

### 4.8. Proteomic Screening for p27-Interacting Factors

HEK293 cells were transfected with 10 µg each of pCS2+HAp27 wild type, pCS2+HAp27 RXL mutant (RXL motif amino acids mutated 30RNL32 → 30AAA32), or pCS2 + p27 wild type (untagged) per 100 mm dish. The cell lysates were mixed with anti-HA agarose beads by rotation at 4 °C overnight. After the beads were rinsed with TBS containing 0.1% Tween 20, immunoprecipitated protein complexes were dissociated from beads with 0.2 M glycine (pH 2.0). The solution containing proteins were neutralized with 1 M Tris-HCl (pH 8.5) and precipitated with final 10% trichloroacetic acid. The pellets were rinsed with 100% acetone, dissolved in TBS, and subjected to SDS-PAGE and CBB staining. HA-specific protein bands were sliced and subjected to liquid chromatography mass spectrometry (LC-MS), which was performed by Y. Kasahara (Hokkaido University).

### 4.9. Yeast Two-Hybrid Assay

A yeast two-hybrid assay was carried out, according to the method previously described [40]. The cDNAs of the candidate p27-interacting factors derived from U-2 OS cells were cloned into pGAD424, and p27 wild-type and RXL mutant cDNAs were cloned into pGBTK. These pGAD424 and pGBTK plasmids were introduced into yeast strains PJ69-4A and PJ69-4Aα [41], respectively, and the transformants were mated in designated bait-prey pairs, which were spotted onto selection plates and incubated for 5 days for the interaction specificity test.

### 4.10. RNA Interference

The NPM1-targeting shRNA vectors were constructed by inserting the following oligonucleotides [42] into pSIREN RetroQ: shNPM1 No. 1, 5′-GCGCCAGTGAAGAAATCTATA-3′ and shNPM1 No. 2, 5′-CCTAGTTCTGTAGAAGACATT-3′. The control shRNA used was a negative control shRNA (Takara bio Inc.) provided with pSIREN RetroQ. These vectors were retrovirally transferred, as described above, into the U-2 OS Tet-on p27-EGFP and HT1080 Tet-on p27-EGFP cells, and NPM1 knockdown efficiency was measured by Western analyses.

### 4.11. Mouse Xenograft Model

Five-week-old male BALBnu/CrlCrlj nude mice (Charles River, Wilmington, MA, USA) were used for the subcutaneous injection of HT1080 cells carrying Dox-inducible p27 and shNPM1 (or control shRNA). Then, 1 × 10^6^ cells were suspended in 0.1 mL cold 50% DMEM and 50% Matrigel (Corning, Corning, NY, USA), and subcutaneously injected into back of nude mice. One week after injection, nude mice were fed with or without 200 µg/mL doxycycline in drinking water and the tumor size was measured every day. The tumor volume was calculated as follows: volume (mm^3^) = (length) × (width)^2^ × 0.523. Seventeen days later, the mice were sacrificed, and the tumors were excised. All animal experiments were performed under the approval of the Tokyo University of Agriculture Animal Experiment Committee.

### 4.12. Immunofluorescence of Tumors

Excised tumors were fixed with 3.7% formaldehyde in PBS at 4 °C for 24 h. Fixed tumors were dehydrated with 30% sucrose in PBS at 4 ºC, for 48 hours. After tumors were embed with Tisse-Tek O.C.T. Compound (Sakura Finetek, Tokyo, Japan), they were sliced by Cryostat (12 µm thick). Sections were refixed with 3.7% formaldehyde in PBS for 15 minutes at room temperature, washed three times with PBS, treated with blocking buffer (2% goat serum and 0.3% Triton X-100 in PBS) for 1 hour at room temperature, and incubated with mouse anti-NPM1 monoclonal antibody (FC-61991, Thermo Fisher Scientific) overnight at 4 °C. After washed three times with PBS, sections were incubated with anti-mouse IgG-Alexa Fluor 555 secondary antibodies (Cell Signaling Technology, Danvers, MA, USA) for 1 hour at 4 °C, washed three times with PBS, counterstained with 0.2 µg/mL DAPI in H_2_O for five minutes, washed once with H_2_O, and mounted with Fluoro-KEEPER (Nacalai Tesque, Kyoto, Japan).

### 4.13. Quantification and Statistical Analysis

Data are representative of at least three independent experiments, as indicated. All results are expressed as mean (± SEM). Statistical significances of differences among data groups were analyzed by the Student’s *t*-test.

## 5. Conclusions

In general, it is known that protein levels of CDK inhibitor p27 are often low and its functions are suppressed mainly by protein degradation in cancers. However, in this study, we found that some cancer cells, excluding normal cells, that overexpress p27 continue proliferation. Thus, the antiproliferative p27 function is qualitatively, not quantitatively, suppressed in those cancer cells. We identified NPM1, which is highly expressed in cancer cells, as a novel factor interacting with p27 and confirmed that the increased expression of NPM1 in cancer cells suppresses p27 function and promotes cell proliferations in vitro and in vivo. We suggest the mechanism as a novel mechanism of suppressing p27 function and NPM1 is a potential target for cancer treatment to recover p27 function.

## Supplementary Materials

The following are available online at https://www.mdpi.com/2072-6694/12/10/2886/s1, Figure S1: NPM1 is specifically co-immunoprecipitated with p27, Figure S2: Cell number counts used for calculating the growth rates in Figure 3C,D, Figure S3: In both NIH/3T3 and U-2 OS cells, p27 is localized predominantly in the nucleus, Figure S4: Immunofluorescence analysis of the co-localization of NPM1 and p27 in mammalian cells.
