# Supplementary material for: Increased Expression of NPM1 Suppresses p27Kip1 Function in Cancer Cells
Source: Cancers (Basel). 2020 Oct 8;12(10):2886. doi: 10.3390/cancers12102886 (PMC7600800; doi:10.3390/cancers12102886)
Supplement: Supplementary file 1 [file cancers-12-02886-s001.pdf]

Supplementary Materials

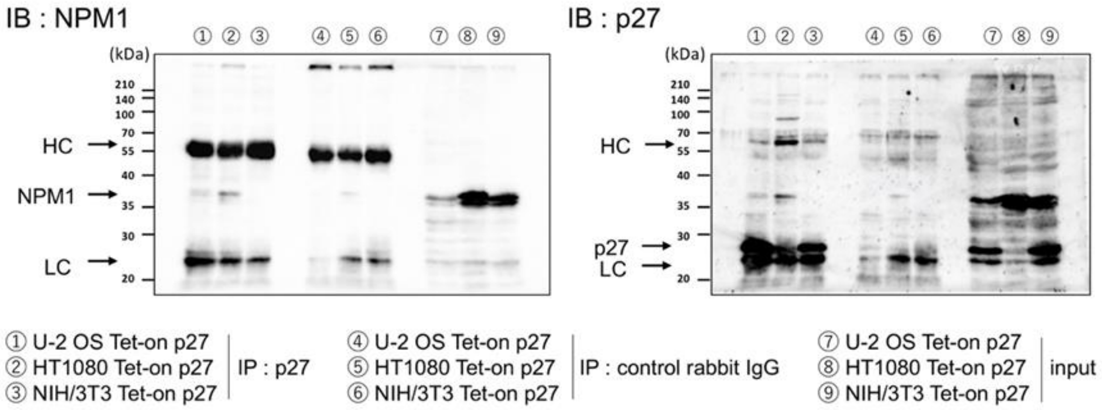

Figure S1. NPM1 is specifically co-immunoprecipitated with p27. U-2 OS, HT1080, and NIH/3T3 cells carrying Dox-inducible p27 were treated with 10 μg/mL Dox, for 24 h before harvest. The cell lysates were subjected to immunoprecipitation using anti-p27 rabbit polyclonal antibody (lanes 1–3) or control rabbit IgG (lanes 4–6) and Western blot analysis using rabbit anti-NPM1 (left) and anti-p27 (right) polyclonal antibodies. HC and LC, IgG heavy chain and light chain, respectively.

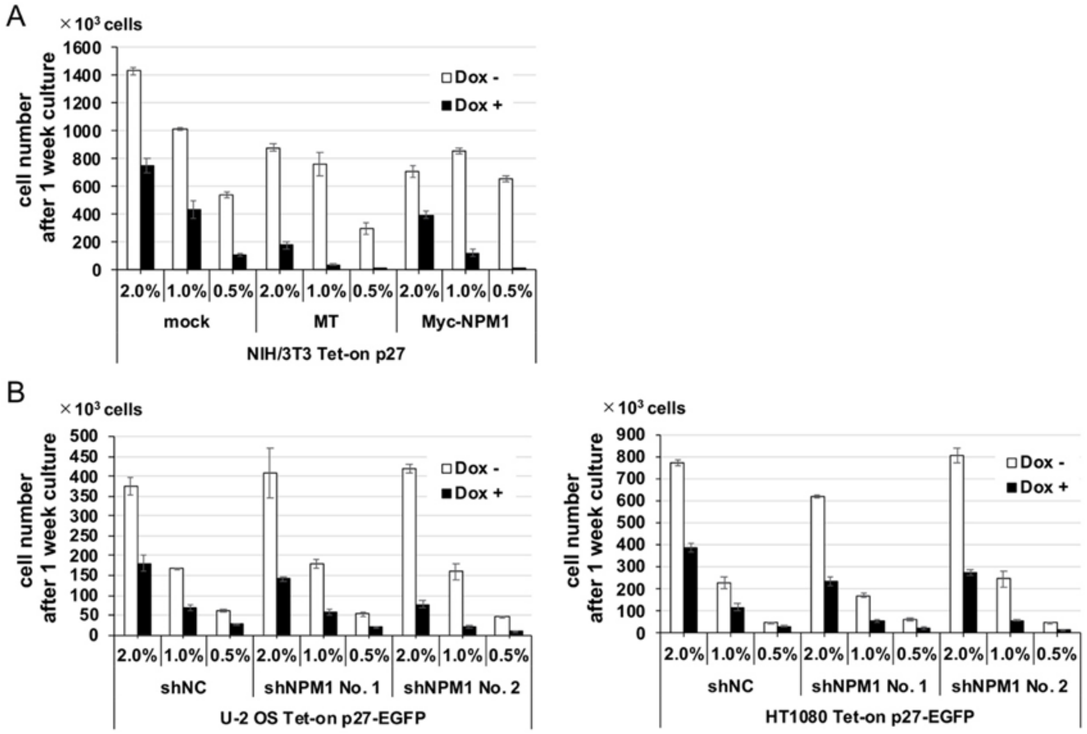

Figure S2. Cell number counts used for calculating the growth rates in Figure 3C,D. (A) NIH/3T3 Tet-on p27 cells with or without NPM1 overexpression in Figure 3C; (B) U-2 OS (left) and HT1080 (right) Tet-on p27-EGFP cells with or without NPM1 knockdown in Figure 3D. The cells were seeded at low densities (1% 3 × 10^{3} cells per 35 mm dish), cultured for one week, with or without 10 μg/mL doxycycline (Dox), and counted.

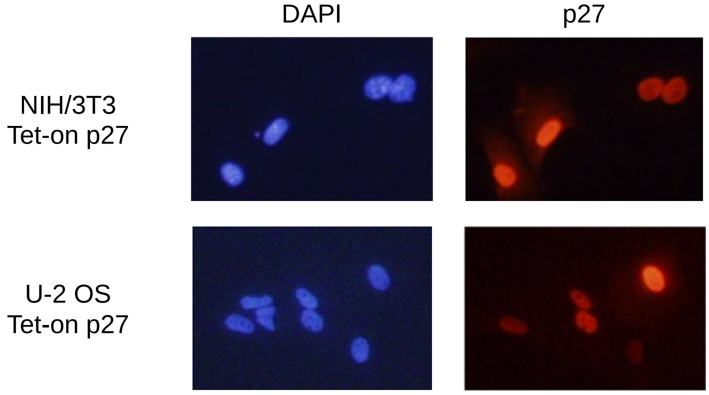

Figure S3. In both NIH/3T3 and U-2 OS cells, p27 is localized predominantly in the nucleus. NIH/3T3 and U-2 OS cells carrying Tet-on p27 were treated with 10 μg/mL Dox, for 24 hours before fixing, and then subjected to immunofluorescence analysis using rabbit polyclonal anti-p27 antibody (C-19, Santa Cruz Biotechnology). Nuclei were stained with DAPI.

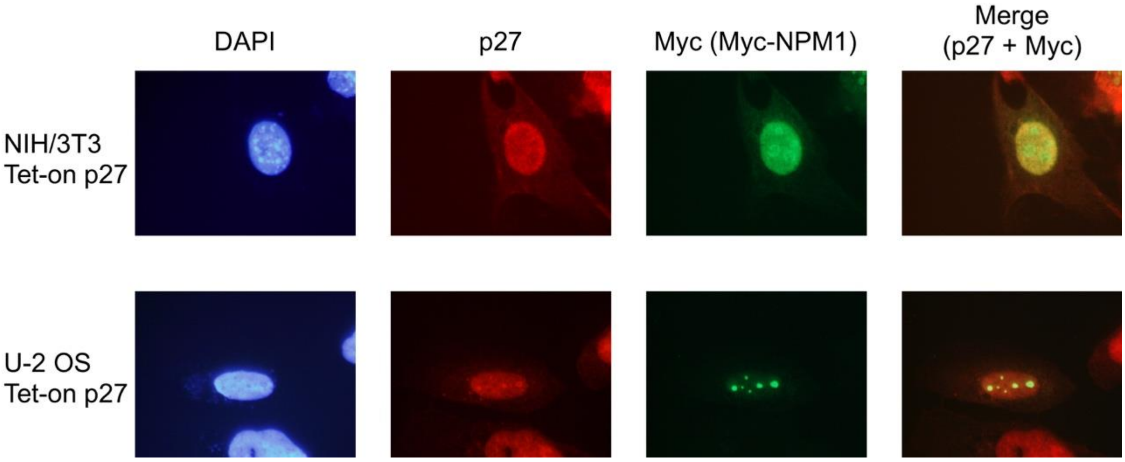

Figure S4. Immunofluorescence analysis of the co-localization of NPM1 and p27 in mammalian cells. U-2 OS and NIH/3T3 cells carrying the Tet-on p27 were transfected with Myc-NPM1 and treated with doxycycline for 24 h before fixing, and then subjected to immunofluorescence analysis using mouse monoclonal anti-Myc and rabbit polyclonal anti-p27 antibodies.
